# Three-dimensional nanometre localization of nanoparticles to enhance super-resolution microscopy

**DOI:** 10.1038/ncomms8764

**Published:** 2015-07-27

**Authors:** Pierre Bon, Nicolas Bourg, Sandrine Lécart, Serge Monneret, Emmanuel Fort, Jérôme Wenger, Sandrine Lévêque-Fort

**Affiliations:** 1 Laboratoire Photonique Numérique et Nanosciences (LP2N), CNRS UMR5298, Institut d'Optique Graduate School, Bordeaux University, Rue Francois Mitterand, 33400 Talence, France; 2 Institut Langevin, ESPCI ParisTech, CNRS UMR 7587, PSL Research University, 1 rue Jussieu, Paris 75238, France; 3 Institut des Sciences Moléculaires d'Orsay (ISMO), University Paris-Sud, CNRS UMR 8214, Orsay 91405, France; 4 Centre de photonique Biomédicale (CPBM/CLUPS/LUMAT) FR2764, University Paris-Sud, Orsay 91405, France; 5 CNRS, Aix Marseille Université, Ecole Centrale Marseille, Institut Fresnel UMR7249, 13013 Marseille, France

## Abstract

Meeting the nanometre resolution promised by super-resolution microscopy techniques (pointillist: PALM, STORM, scanning: STED) requires stabilizing the sample drifts in real time during the whole acquisition process. Metal nanoparticles are excellent probes to track the lateral drifts as they provide crisp and photostable information. However, achieving nanometre axial super-localization is still a major challenge, as diffraction imposes large depths-of-fields. Here we demonstrate fast full three-dimensional nanometre super-localization of gold nanoparticles through simultaneous intensity and phase imaging with a wavefront-sensing camera based on quadriwave lateral shearing interferometry. We show how to combine the intensity and phase information to provide the key to the third axial dimension. Presently, we demonstrate even in the occurrence of large three-dimensional fluctuations of several microns, unprecedented sub-nanometre localization accuracies down to 0.7 nm in lateral and 2.7 nm in axial directions at 50 frames per second. We demonstrate that nanoscale stabilization greatly enhances the image quality and resolution in direct stochastic optical reconstruction microscopy imaging.

## Abstract (web summary)

Tracking and stabilizing sample drifts is crucial towards realizing nanometer resolution in superresolution microscopy; metal nanoparticles can provide drift information but diffraction remains a challenge. Here, Bon *et al*. combine intensity and phase information to reach three-dimensional subnanometre accuracies.

## Graphical abstract

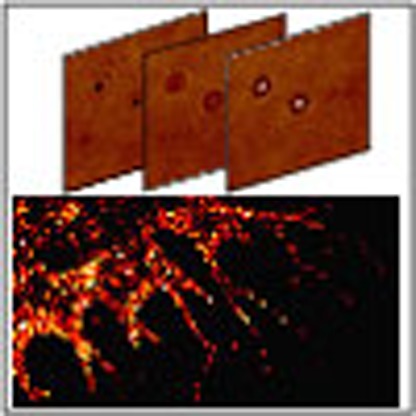

Super-resolution microscopy and nanoscopy techniques are pushing the limits of optical imaging down to the molecular scale^1^,^2^. Reaching this ultimate resolution requires stabilizing the whole set-up against mechanical and thermal drifts with an even better spatial accuracy and a fast reaction time. This forms a major technical challenge, especially for pointillist super-resolution approaches such as (f)PALM^3^,^4^ or (d)STORM^5^,^6^,^7^ that require multiple image acquisitions and long integration times. Although various approaches have been proposed to tackle this problem^5^,^8^,^9^,^10^,^11^,^12^,^13^,^14^, severe restrictions limit the stabilization accuracies, which either lack sensitivity^8^, have a narrow range^8^,^9^,^10^,^11^,^14^, do not compensate for the axial drift^5^,^13^, need a perfectly calibrated sample and objective^12^ or induce background noise^5^,^13^,^14^.

Currently, the most widely used approach involves imaging a fluorescent or scattering nanoparticle to determine the centroid of its point spread function and quantify the sample drifts^15^. While this achieves localization precisions <10 nm in the transverse *xy* dimensions^16^, localization of nanoparticles along the optical axis is much more challenging as diffraction imposes large depths-of-fields of several hundreds of nanometres. Therefore near-field optics^17^ or complex optical elements are often introduced to provide better axial localizations. The latter involve a cylindrical lens or deformable mirrors to introduce astigmatism^18^, or use interferometry to achieve axial resolutions of 10–20 nm^19^,^20^,^21^,^22^. Few techniques already reach better than 10 nm accuracy^9^,^10^,^23^,^24^ but they require a double-objective set-up, which significantly complicates the optical alignment and is not suitable to all samples. Moreover their dynamic range is limited and thus they are unable to correct for important defocus. Alternatively, commercial systems track the focus by monitoring the reflection of infrared light at the coverslip surface^25^,^26^,^27^. The axial resolution is a fraction of the depth-of-field, and typically amounts to ≥20 nm. Despite impressive progress of earlier works, breaching below the 10 nm axial resolution level with an important dynamic range and a fast acquisition rate remains a challenge imposing a new thinking of the super-localization approach.

Here we realize super-localization of nanoparticles at video rate with nanometre accuracy along both transverse and axial directions using simultaneous intensity and phase imaging with a wavefront-sensing device. Contrarily to previous techniques relying only on the intensity of light, our approach takes full advantage of the additional phase information. We describe how to exploit the combined intensity and phase response of gold nanoparticles to achieve unprecedented single-shot three-dimensional (3D) localization accuracies of 1.5 nm in lateral and 6.5 nm in axial directions at a fast video rate of 50 frames per second. The technique can guaranty sub-nanometre 3D localization accuracies if longer integration times are considered or if several nanoparticles are tracked simultaneously. The 3D tracking is remarkably robust even in the occurrence of large drifts of several microns, as the knowledge of the full scalar electromagnetic field (amplitude and phase) always allows to numerically retrieve the best focus position. We use the 3D super-localization of nanoparticles to stabilize the microscope during dSTORM imaging. We demonstrate that this full 3D drift correction method performs significantly better than current conventionally used techniques, improving both the dSTORM spatial resolution and the signal-to-noise ratio for single molecule fluorescence detection. As additional advantages of our approach, it is conveniently implemented using a commercial wavefront-sensing device on a lateral port of the microscope, and does not require neither complex interferometric or holographic elements, nor a perfect knowledge of the nanoparticle type or shape. As the process does not rely on fluorescence, the photon flux is readily high enough to enable fast acquisition times below the millisecond range; moreover, as there is no photobleaching of the nanoparticle, the tracking duration is unlimited. As an additional key advantage, the 3D super-localization precision is maintained in the nanometre regime even in the occurrence of large 3D fluctuations up to several microns.

## Results

### Intensity and phase response of nanoparticles on focusing

**Figure 1:**
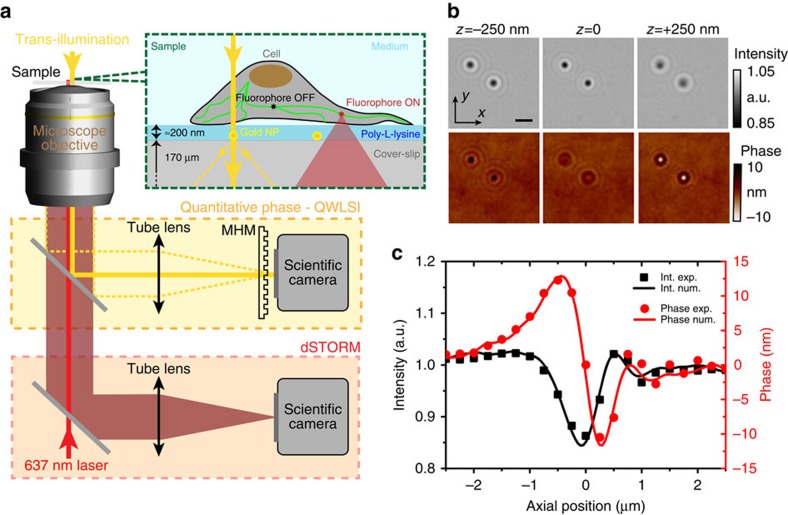
Quantitative intensity and phase imaging to localize nanoparticles. (**a**) Schematic of the optical set-up and sample. The microscope port highlighted in yellow is dedicated to intensity and phase imaging of nanoparticles in transillumination using the microscope lamp, with MHM the acronym for Modified Hartman Mask. The port highlighted in red corresponds to dSTORM imaging. (**b**) Intensity and phase images of two 100 nm gold nanoparticles, in-focus (z=0) or slightly defocused (*z*=±250 nm). Note the contrast inversion in the phase images on defocusing and the weak variations of the intensity signal. The scale bar, 2 μm. (**c**) Intensity (black squares) and phase (red dots) response of a single 100 nm gold nanoparticle recorded versus the mechanical sample displacement. Lines are the results of the numerical propagation computed using the *z*=0 plane data only; they are not numerical fits to the experimental data.

Our stabilization method relies on monitoring the position of a gold nanoparticle using combined intensity and phase imaging with a commercial wavefront-sensing device (Fig. 1a). In the visible and near infrared spectral range, the imaginary part of the complex refractive index of gold dominates over the real part^28^. Therefore, when a gold nanoparticle is perfectly set at the microscope focus (Fig. 1b; *z*=0), the intensity drop is large as light is lost due to absorption and scattering^29^,^30^, and the phase response is weak as the optical retardation is almost negligible for a subwavelength nanoparticle. The intensity image at the focus provides 2D super-localization of the nanoparticle in the transverse plane using a conventional 2D Gaussian fit of the diffraction-limited spot^31^,^32^, as reported for fluorescence emitters in earlier works^33^. However, in the case of a nanometre axial defocus (Fig. 1b,c), the intensity barely changes. Moreover, the intensity dependence on the axial position is symmetric respective to the optimal focus position. Axial super-localization is therefore difficult to achieve using only the intensity images without time-consuming physical displacement of the sample stage and acquisition of multiple intensity images.

An essential element in our technique is that the phase response (or equivalently the optical retardation) varies sharply with the nanoparticle axial position, and bears a sign inversion respective to the latter (Fig. 1b,c). The slope of the phase response versus the axial defocus *z* directly scales with the intensity attenuation (see Supplementary Discussion for a demonstration). Therefore the phase slope versus the axial defocus can be very significant in the case of absorbing metal nanoparticles despite the small real part of the refractive index of gold and the sub-resolution size of the nanoparticle. This phase information is the key to reach super-localization with nanometre accuracy along the axial direction.

Imaging both in phase and intensity provides a complete representation of the scalar electromagnetic field for a fixed axial *z* position. This information enables us to numerically compute the effect of propagation in any arbitrarily chosen plane (see Supplementary Methods for details). As seen in Fig. 1c, the results of the numerical propagation agree remarkably with the experimental data, even for large defocuses over 2.5 μm (six times the depth-of-field). Using only a single measurement of the couple intensity-phase images for any (unknown) observation plane, we can compute the 3D displacement to the best focus position with nanometre accuracy without moving any optomechanical element.

### Nanometre localization accuracy of gold nanoparticles

**Figure 2:**
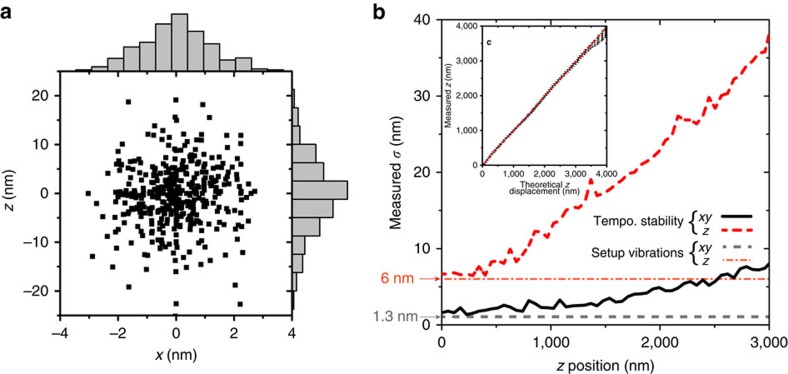
Nanometre super-localization accuracy of a 100 nm gold nanoparticle. (**a**) Scatter plot of the nanoparticle lateral *x* and axial *z* positions showing 400 measurements at 50 frames per second video rate (20 ms acquisition time per measurement, the numerical computation time is negligible). The *x* and *z* position histograms are represented in grey. (**b**) s.d. of the measured nanoparticle position along *x* (black line) and *z* (red dashed line) as a function of the calibrated axial defocus induced by the piezo stage. For each axial stage position, 100 measurements are taken at 50 Hz to estimate the s.d. of the localization. The noise level corresponding to the set-up vibrations over the 2 s integration time is represented by a grey dashed line for the *x* direction and by an orange dashed dot line for the *z* direction. (**c**) Measured axial position as a function of the calibrated piezo stage *z* displacement.

We quantify the accuracy of our 3D super-localization technique using 400 repeated position measurements at 50 Hz video rate (20 ms acquisition plus processing time per measurement). Figure 2a displays a scatter plot of the lateral *x* and axial *z* positions. The standard deviation (s.d.) of the position measurement at 50 Hz acquisition rate is *σ*(*p*_*xy*_)=1.5 nm in the lateral directions and *σ*(*p*_*z*_)=6.5 nm in the axial direction. This corresponds to remarkable super-localization accuracies of ∼*λ*/400 along *xy* and ∼*λ*/90 along *z* direction.

An important parameter is the extent of the axial range where the super-localization procedure holds. To do this, we apply calibrated displacements to the nanoparticle sample with a 3D piezo stage while recording the nanoparticle position (Fig. 2b,c). Remarkably, even for very large defocus up to 3 μm (equivalent to 7.5 times the imaging depth-of-field set by diffraction), the lateral and axial accuracies can be better than 10 nm and 40 nm, respectively, while working at 50 frames per second. To reach such values, our approach takes full advantage of the phase information which enables the numerical computation of the propagation and refocusing (see Supplementary Methods and Supplementary Movie 1).

We quantify the accuracy that is intrinsic to our super-localization approach by monitoring the correlated displacement of two immobilized nanoparticles within the field-of-view (see Supplementary Methods for a mathematical description). At 50 frames per second we measure a s.d. of 0.7 nm and 2.7 nm in the lateral and axial direction, respectively. These results can be further improved by an arbitrary 
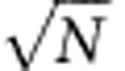
 factor while using longer integration times and averaging *N* images. This illustrates the potential to reach sub-nanometre localization accuracies in 3D.

### dSTORM enhancement by drift compensation at the nanoscale

Thermal and mechanical drifts must be actively compensated to get super-resolution imaging techniques to their maximum resolution. We perform dSTORM imaging of fixed chinese hamster ovary (CHO) cells using the super-localization information from 100 nm gold nanoparticles to refocus the sample and compensate for 3D drifts. The nanoparticles are fixed on the coverslip substrate by a poly-L-lysin (PLL) layer on which the cells are cultivated (Fig. 1a). The sample preparation and the dSTORM imaging reconstruction are described in the Methods section with additional details in the Supplementary Methods.

**Figure 3:**
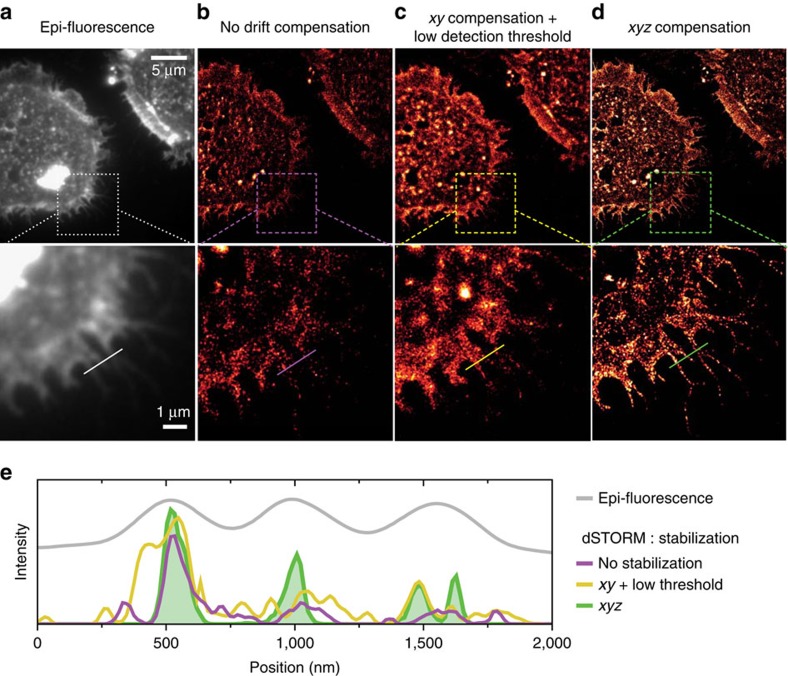
Super-localization enhanced dSTORM imaging on actin phalloidin-A647 labelling. (**a**) Epi-fluorescence image. (**b**) dSTORM image without drift correction. (**c**) dSTORM reconstruction with lateral (*x*, *y*) drifts stabilization. The detection threshold is lowered to maximize the number of detected molecules and thus reduce the negative effects of axial drifts. (**d**) dSTORM reconstruction with full 3D drift correction. (**e**) Intensity cuts along the lines in the sub-images (**a**–**d**). The total acquisition time is 25 min.

Figure 3 shows dSTORM images of F-actin networks in fixed CHO cells labelled by Alexa Fluor 647 phalloidin (more dSTORM images on tubulin network are shown in the Supplementary Fig. 1). When no stabilization is active to compensate for spatial drifts (Fig. 3b), very few fluorescent molecules are detected and the spatial resolution is poor. A conventional approach to recover the detection events in the case of defocus is to lower the fluorescence detection threshold. The lateral (*x*, *y*) drifts can be compensated using the intensity images from the gold nanoparticles fiducials. However, in that case (Fig. 3c), the localization signal-to-noise ratio for each fluorophore is low, hence the reconstruction uncertainty is relatively large and the dSTORM resolution is limited. In the case of the full 3D stabilization using our method (Fig. 3d), the resolution gain is clearly visible as sub-networks are appearing on the reconstructed images. We estimate the dSTORM spatial resolution from localization histograms (Fig. 3e and Supplementary Fig. 2). The lateral resolution for the F-actin experiments with 3D stabilization is 11 nm for the smallest fibres, realizing a threefold improvement of the resolution obtained without lateral drift compensation (see Supplementary Fig. 2 and Supplementary Table 2).

### Comparison with conventional stabilization approach

**Figure 4:**
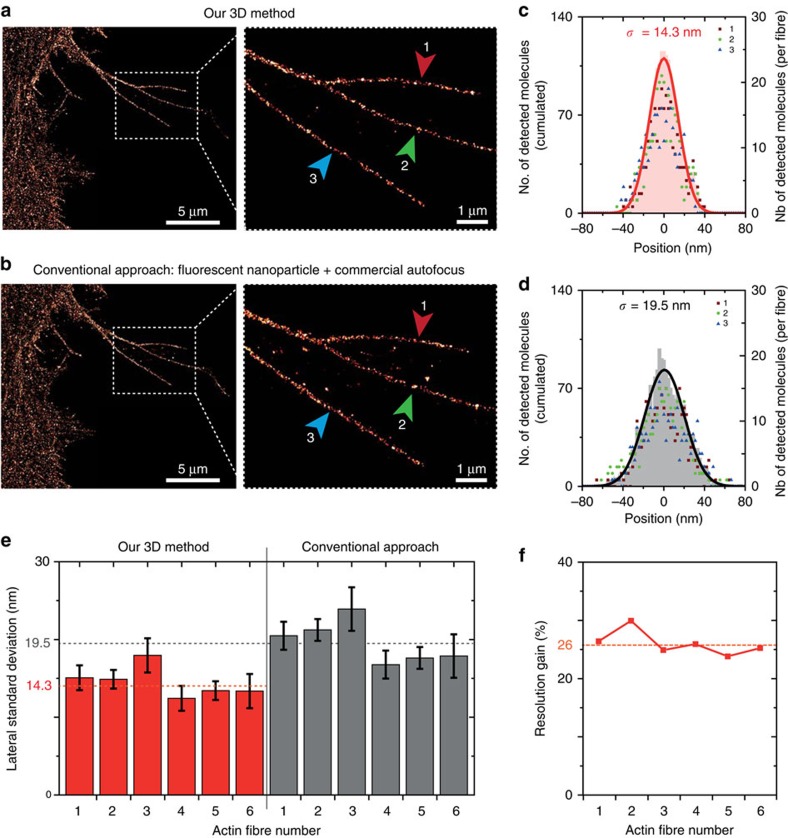
dSTORM image comparison and resolution improvement between our approach and a conventional technique. (**a**) Image of labelled F-Actin in CHO cells obtained using our 3D stabilization technique. The insert shows the fibres used for assessing the dSTORM resolution (indicated by the arrows 1, 2 and 3). (**b**) Same as **a** using a conventional drift correction technique combining a commercial autofocus (Nikon Perfect Focus) and 2D lateral stabilization using fluorescent nanoparticle tracking (artificial star). (**c**,**d**) Histograms of the molecule positions deduced from the data in **a**,**b**, respectively. The number of detected events is similar in both cases. Data points correspond to the individual fibres highlighted in the inserts in **a**,**b**, the shaded area denotes the cumulated data and the line is a Gaussian fit used to determine the localization s.d. *σ*. (**e**) s.d. found for the different fibres using the two methods. The fibres 1–3 are shown in **a**,**b**, the fibres 4–6 are displayed in Supplementary Fig. 3. The dashed horizontal lines indicate the average value found for each method, demonstrating a better resolution with our method. (**f**) dSTORM resolution improvement computed for each actin fibre as the ratio of the s.d. values in **e**. An average gain of 26% is obtained.

To appreciate the benefit of our technique, we perform a side-by-side comparison with a conventional approach combining an active axial autofocus system (Perfect Focus, Nikon, Japan) and lateral drift compensation using a fluorescent nanoparticle imaged on the fluorescence camera. Importantly, the comparison is performed on the same sample under equivalent dSTORM imaging conditions (see Methods section for details). Figure 4 and Supplementary Fig. 4 shows crisper images using our approach. To quantify the dSTORM resolution with each method, we compute the s.d. found for the localization histograms along the same set of F-actin fibres. For each fibre, the s.d. is lower with our method than with the conventional approach (Fig. 4e). This corresponds to a remarkable improvement of the dSTORM spatial resolution by 26% with our approach (Fig. 4f). This result demonstrates the superior performance of our technique, which does not noticeably depend on the actin fibre being considered.

**Figure 5:**
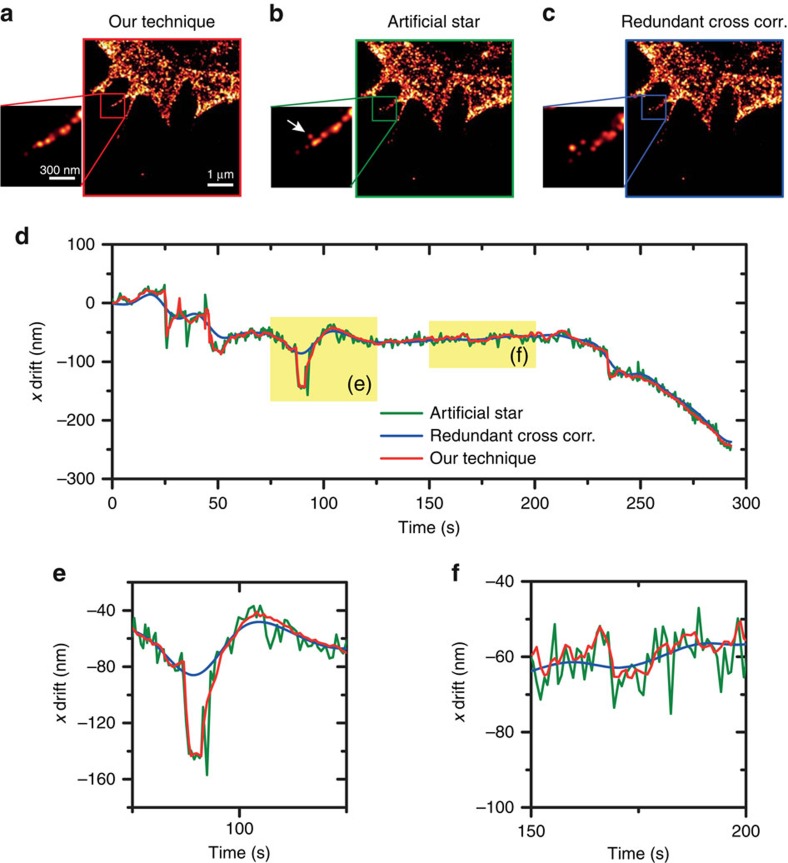
Improved dynamic lateral accuracy of our approach as compared with artificial star and redundant cross-correlation. For this experiment, the axial stabilization is performed with our approach. (**a**–**c**) dSTORM images of F-actin in fixed CHO cells. (**a**) Drift compensation using our technique. (**b**) Drift compensation by artificial star tracking on each acquired fluorescent image, using a fraction of the dSTORM excitation laser light, back-scattered by the gold nanoparticles. (**c**) Numerical *a posteriori* drift correction by redundant cross-correlation (subset of 50 images). (**d**) Temporal evolution of the drift measured along the *x* direction. (**e**) Zoom-in on **d** between 75 and 125 s showing the lack of accuracy for the redundant cross-correlation approach in the case of fast events. (**f**) Zoom-in on **d** between 150 and 200 s showing the larger noise found with the artificial star approach as compared with our method.

In a second comparison, we focus on the temporal performance of our approach to accurately compensate for quick drifts. Here we use the same set of dSTORM F-actin images in the presence of 100 nm gold nanoparticles, and implement the ‘artificial star' method by selecting a fluorescence emission filter transmitting a small amount of the back-scattered excitation laser light to get a fixed spot on each dSTORM image. We also evaluate drift correction by redundant cross-correlation, which is a purely numerical *a posteriori* treatment^34^. For this set of measurements, the axial drift is compensated with our technique as we focus on the correction of lateral drifts. Comparing the dSTORM images compensated with our approach (Fig. 5a) and the artificial star (Fig. 5b) reveals that some molecules are miss-located with the artificial star (such as the one indicated with the white arrow). The redundant cross-correlation algorithm leads to dSTORM images with a blurrier look, especially on thin structures (Fig. 5c). Monitoring the temporal dynamics of the lateral drifts provides additional insights (Fig. 5d–f). The redundant cross-correlation algorithm is unable to reconstruct quick drifts, leading to a smoother evaluated drift. The artificial star provides results that are comparatively significantly noisier. Altogether, these results show that our technique provides better performance in both accuracy and temporal dynamics over the two conventional methods. A comparison with a commercial autofocus system monitoring the reflection of infrared light at the coverslip surface is provided in the Supplementary Fig. 4. Again, the results confirm the better spatial and temporal accuracy with our method.

To complete the discussion, we show in the Supplementary Discussion that the optical aberrations have a negligible effect on our observations and that any nanoparticle in the field-of-view can be used to track the sample drifts. To maximize the optical response of the gold nanoparticles and optimize the sensitivity of our method, it is advantageous to select an illuminating wavelength close to the local surface plasmon resonance of the nanoparticle (see Supplementary Discussion). However, it is noteworthy that our technique is not limited to absorbing metal nanoparticles: dielectric subwavelength objects can also be super-localized in 3D (Supplementary Discussion shows a comparison between gold and polystyrene nanoparticles).

## Discussion

In summary, using the complementary intensity and phase images of light scattered by a metal nanoparticle, we achieve a nanoparticle localization precision of 1.5 nm in lateral and 6.5 nm in axial direction at a video rate of 50 frames per second. The accuracy can be pushed further to 0.7 × 0.7 × 2.7 nm^3^ while keeping the same temporal resolution by tracking two nanoparticles to get rid of the vibrations from the piezo stage and camera. The use of endogenous particles can also be considered to simplify the sample preparation: for example, a fixed vesicle inside the cell can be a good probe. This single-shot 3D nanoscale super-resolution is combined with dSTORM fluorescence imaging to enhance the image quality, improving both the spatial resolution and the signal-to-noise ratio. Our approach has several specific advantages. It is implemented only using a commercial wavefront-sensing device on a lateral port of the microscope: there is no complex holographic apparatus or moving elements; it is suitable to any microscope without major modifications, and the illumination wavelength can be easily tuned using regular filters to meet the user's requirements. As the method does not require a laser illumination, there is no speckle noise on the images. As it is not a fluorescent imaging approach, there is no photobleaching and the nanoparticle localization can be very fast with a large number of photons. Moreover, our approach can be used to stabilize super-resolution imaging systems when the sample is embedded in refractive-index-matching medium, as for in-depth STED imaging. Last, knowing the full scalar electromagnetic field allows to compute the propagation back to the best focus plane. This maintains an excellent super-localization precision even with a large and unknown defocus of several microns.

## Methods

### Optical set-up

All measurements are performed on a commercial inverted microscope (TiE, Nikon) equipped with a × 100 numerical aperture=1.49 objective and a double-stage filter set to allow simultaneous nanoparticle imaging at *λ*=594±20 nm (yellow part on Fig. 1a) and fluorescence imaging (red part on Fig. 1a) of Alexa Fluor 647 fluorophores. The intensity and phase imaging is obtained with a commercial quadriwave lateral shearing interferometer (QWLSI)^35^,^36^ (SID4-element, Phasics, France) and a sCMOS camera (Orca Flash 4, Hamamatsu, Japan). The transillumination for phase and intensity sensing is generated by the native microscope Kohler illumination, with an aperture diaphragm closed to the minimum to ensure a beam spatial coherence and a bandpass filter *λ*=594±20 nm tuned with the NP plasmon resonance. The fluorescence is excited by a 637 nm laser (Obis, Coherent, USA) and recorded on a sCMOS camera (Neo, Andor, Ireland). We also used for some experiments a 405 nm laser (Obis) in addition to the 637 nm laser to reactivate the fluorescence blinking.

### NP super-localization from electromagnetic field measurements

A homemade Labview software has been implemented for intensity/phase retrieval, NP super-localization and numerical focusing. The intensity and phase retrieval from the QWLSI camera image is achieved in the Fourier space by using the algorithm described in refs 35, 37. The NP lateral super-localization algorithm is based on nonlinear Levenberg–Marquardt fit by a Gaussian on the intensity image^32^, as commonly done in PALM/(d)STORM^33^. The axial super-localization is obtained by comparing the intensity and phase value in the centre of the NP image to a calibration curve obtained by numerical propagation in different axial planes of one NP electromagnetic field measurement (as displayed in Fig. 1c). The calibration process is made only one time at the experiment beginning and is valid while the particle stays within the field-of-view. In the occurrence of axial defocus, we use numerical propagation to numerically refocus the nanoparticle before performing lateral localization.

### dSTORM imaging samples

We prepared the sample using 1/200 diluted solution of commercial 100 nm gold nanoparticles NP (BBI Solutions, UK). A 200 μl droplet of colloidal solution was deposited and left drying overnight on a cleaned 1.5H coverslip (VWR, Radnor). A droplet of PLL at 0.1% was then deposited on the sample for 1 hour 20 minutes at ambient temperature before washing with distilled water. It ensures both NP immobilization and separation with biological samples by adding an ≈200 nm layer of polymerized PLL^38^. As PLL is biocompatible and stable at 37 °C, cells can be directly plated on the sample. As the NP are isolated by polymerized PLL, there is no NP internalization within the cells. Although the NP-embedded coverslip preparation is easy to obtain, it is noteworthy that commercial coverslips with such fiducial gold particles exist^39^. For dSTORM imaging, CHO cells have been used on the NP prepared coverslips; the culture protocol, staining and labelling are presented in the Supplementary Methods. The fixed samples are mounted in a 50/50% PBS/Vectashield (Vector Laboratories, USA) mixture or a mixture of thiol+oxygen scavenger detailed below. The Vectashield mixture produces Alexa Fluor 647 blinking at very high brilliance^40^ while the thiol+oxygen scavenger mixture allows fluorophore reactivation using near-ultraviolet light^7^.

### Comparison with conventional drift correction techniques

To compare our results with images obtained using state-of-the-art conventional drift correction techniques, we use a fixed fluorescent nanoparticle as an ‘artificial star' to compensate for lateral drifts, together with a commercial autofocus approach (Perfect Focus) to track axial defocus. The fluorescent nanoparticles of 20 nm diameter (FluoSphere 580/605, Life technologies, USA) are chosen to minimize photobleaching and avoid the saturation of the fluorescence camera. The same fixed CHO cell with F-actin labelled by phalloidin-Alexa Fluor 647 is imaged sequentially using first the conventionally used 3D drift compensation technique (Fig. 4a–c, total acquisition time: 15 min) and then with our 3D stabilization approach (Fig. 4d–f, total acquisition time: 17 min). For comparison purpose, the total number of detected molecules is equal in both images. It is possible to make this double imaging procedure with a buffer composed of thiol+oxygen scavenger and a 405 nm laser^7^. The buffer contained a 50 mM concentration of Beta-mercaptoethylamine (MEA, Sigma-Aldrich) and an oxygen scavenger composed of 0.5 mg ml^−1^ glucose oxidase (G0543, Sigma-Aldrich), 40 μg ml^−1^ catalase (C40, Sigma-Aldrich) and 10% (w/v) glucose, dissolved in a buffer consisting of 50 mM Tris and 10 mM NaCl (Sigma-Aldrich). This dSTORM buffer allows reversible blinking of the fluorophores in the dark-state level. They can thus be reactivated thanks to ultraviolet light (for example, a 405 nm laser), which allows to image at the same brilliance the same pool of fluorescent probes at least one more time.

## Additional information

**How to cite this article**: Bon, P. *et al*. Three-dimensional nanometer localization of nanoparticles to enhance super-resolution microscopy. *Nat. Commun.* 6:7764 doi: 10.1038/ncomms8764 (2015).

## Supplementary Material

Supplementary Information Supplementary Figures 1-8, Supplementary Tables 1-3, Supplementary Discussion, Supplementary Methods and Supplementary References

Supplementary Movie 1 A numerical refocusing of intensity and phase images, obtained on 100 nm gold nanoparticles embedded in ~200 nm PLL layer, registered with the same setup as in Fig. 1. The defocus varies from −2.8 μm to +3 μm and is generated by moving the microscope objective. On the left, the registered intensity and phase images are presented while on the right the refocused images are shown.
