# Supplementary material for: Three-dimensional nanometre localization of nanoparticles to enhance super-resolution microscopy
Source: Nat Commun. 2015 Jul 27;6:7764. doi: 10.1038/ncomms8764 (PMC4525210; doi:10.1038/ncomms8764)
Supplement: Supplementary Information — Supplementary Figures 1-8, Supplementary Tables 1-3, Supplementary Discussion, Supplementary Methods and Supplementary References [file ncomms8764-s1.pdf]

Supplementary Figures

dSTORM imaging of tubulin network

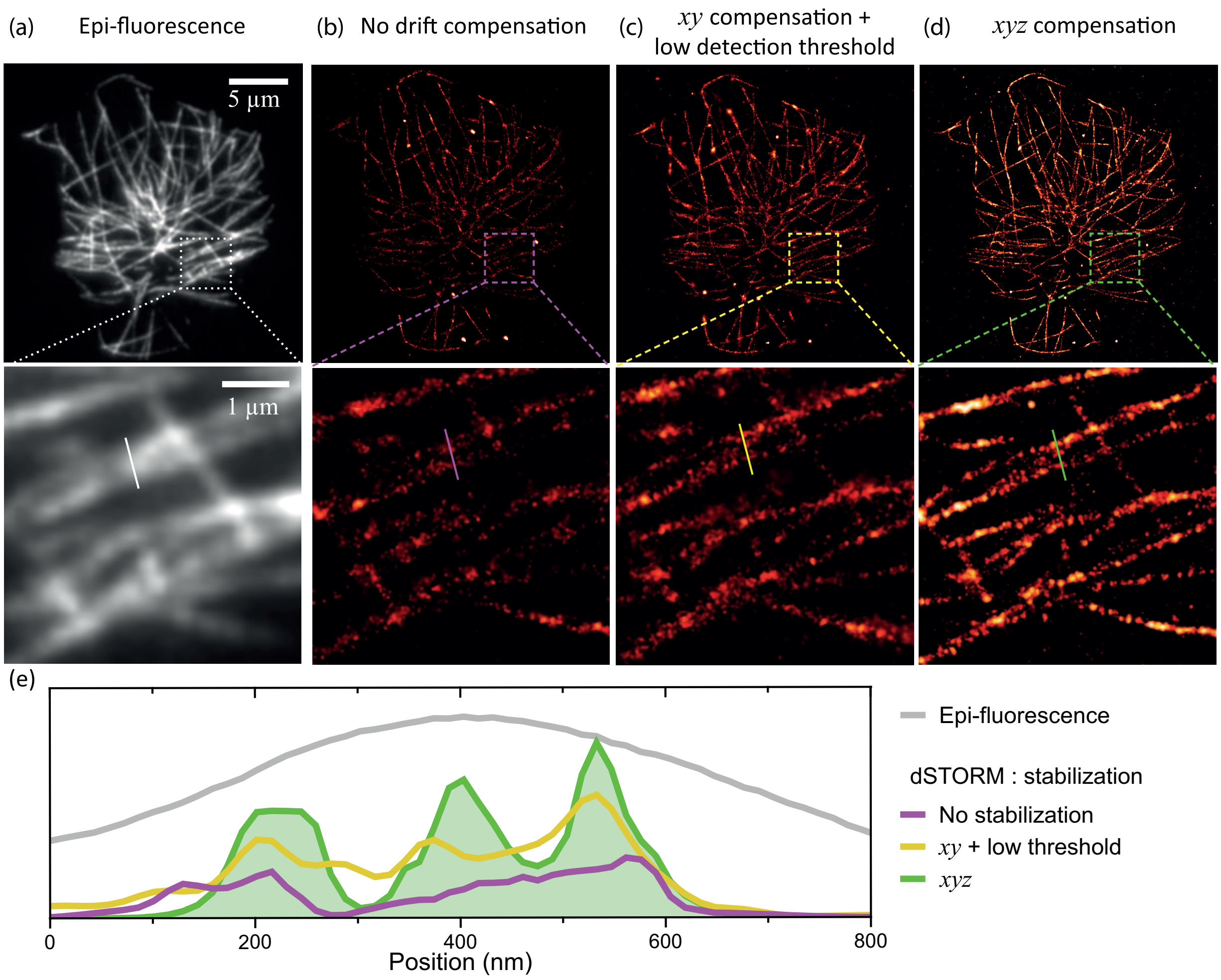

Supplementary Figure 1: **Superlocalization enhanced dSTORM imaging on tubulin immunolabeling**. This figure is similar to Fig. 3 with tubulin labeled by antibody coupled with Alexa Fluor 647 (Total acquisition time: 16 min). (a) Epi-fluorescence image. (b) dSTORM reconstruction simulating the effect of the measured axial z drift that would have been accumulated during the whole experiment if no stabilization had been applied. (c) dSTORM reconstruction with lateral drift stabilization (simulating the axial z drift) with a very low detection threshold τ to maximize the number of detected molecules. (d) dSTORM reconstruction with full 3D drift compensation. (e) Intensity cuts along the lines in subimages *(a-d)*.

Image resolution gain with 3D drift correction

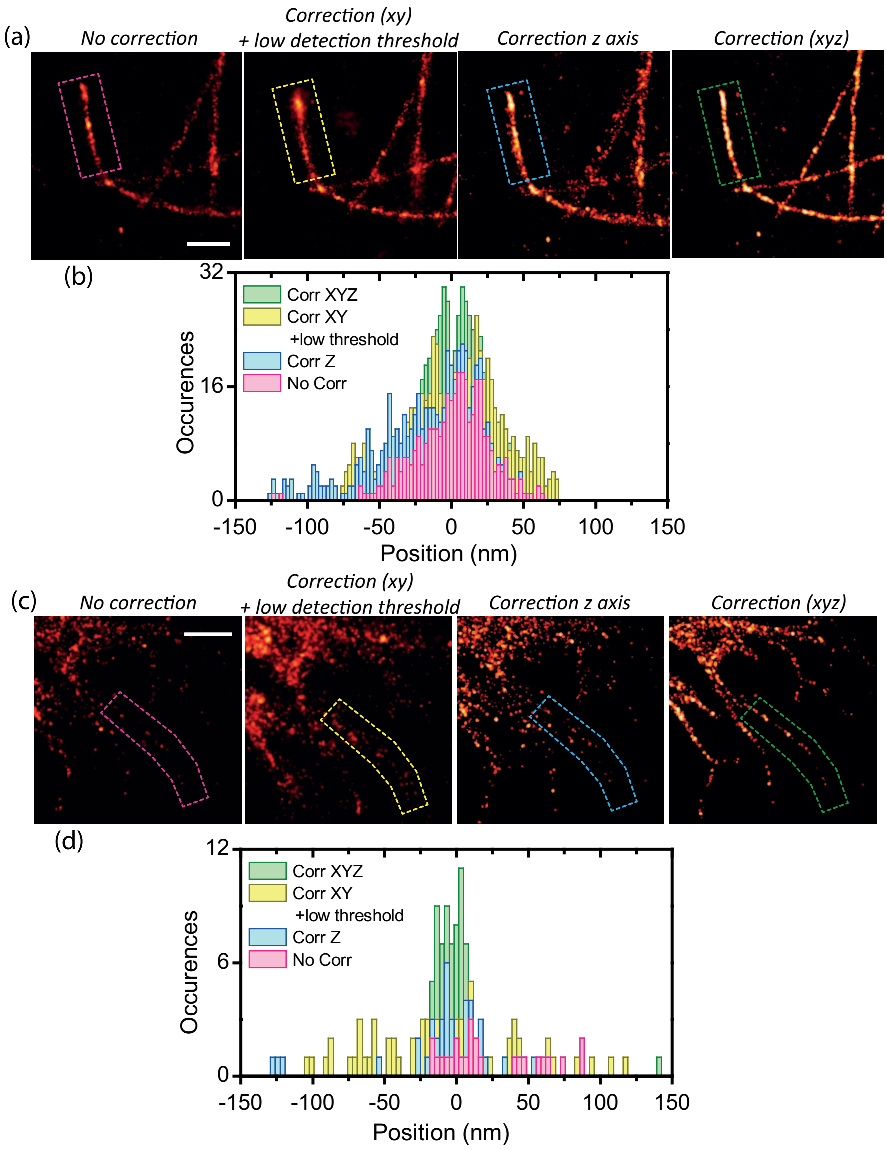

Supplementary Figure 2: (a) Super-resolved images without drift compensation (left), with lateral drift stabilization only and a low molecule detection threshold (middle-left), with axial drift stabilization only (middle-right) and with 3D stabilization (right) of tubulin network labeled with A647 in CHO. The images are extracted from Suppl. Fig. 1. (b) Detection position histogram along the tubulin filament highlighted in *(a)*. In pink, without drift compensation, in blue with axial drift stabilization only, and in green with 3D stabilization. (c) same as *(a)* on F-actin network labeling, extracted from Fig. 3. (d) Same as *(b)* obtained from the F-actin filament highlighted in *(c)*. Scale bars: 1 μm.

Supplementary dSTORM images comparing our method with a conventional technique

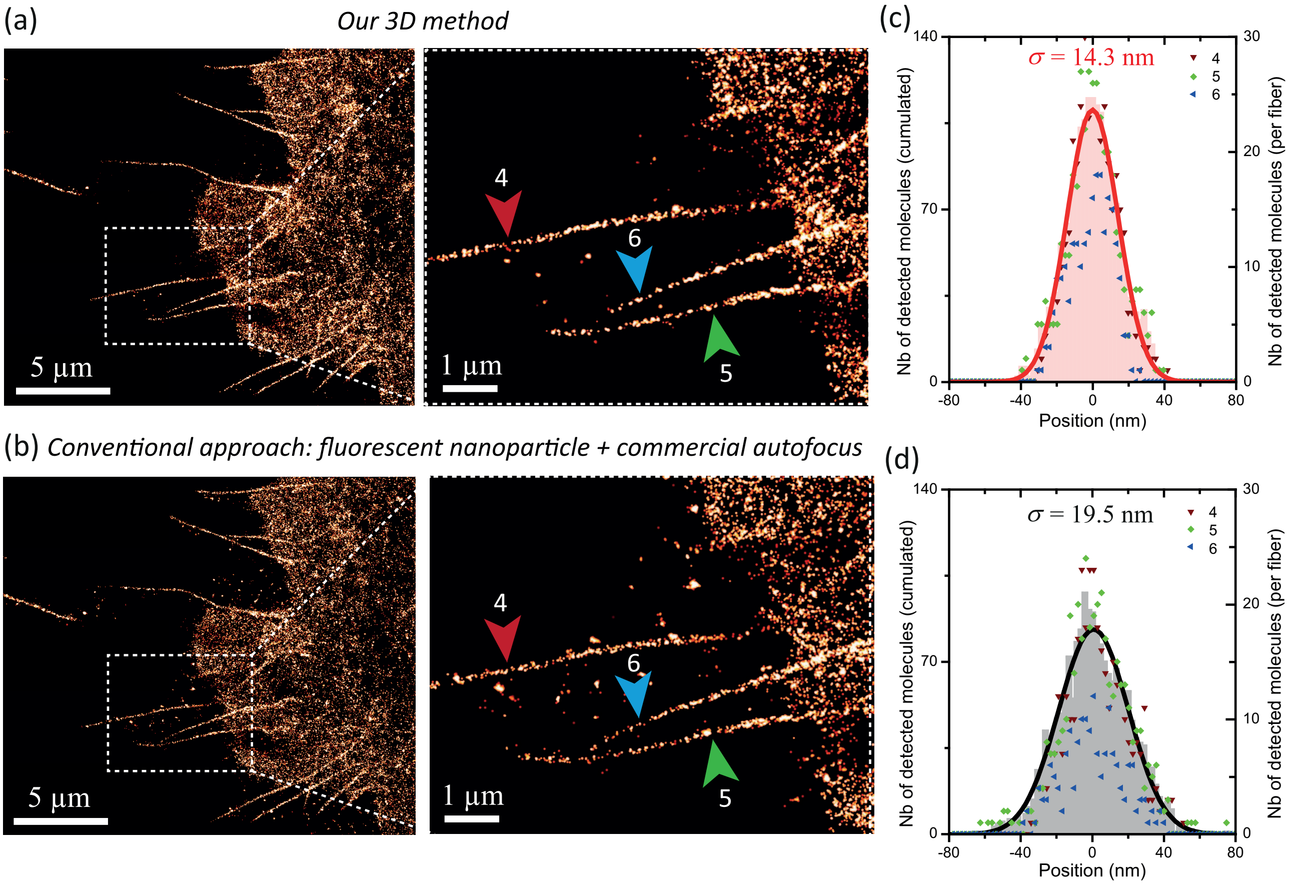

Supplementary Figure 3: Same as Fig. 4 of the main document for a different set of F-actin fibers. The dSTORM images in (a) are obtained using our 3D stabilization technique, the ones in (b) using a conventional drift correction technique combining a commercial autofocus and 2D lateral stabilization using fluorescent nanoparticle tracking. The insert shows the fibers used for assessing the dSTORM resolution in Fig. 4e,f. (c,d) Histograms of the molecule positions deduced from the data in *(a,b)* respectively. The number of detected events is similar in both cases. Data points correspond to the individual fibers highlighted in the inserts in *(a,b)*, the shaded area denotes the cumulated data and the line is a Gaussian fit used to determine the localization standard deviation σ.

Axial localization accuracy as compared to commercial autofocus system

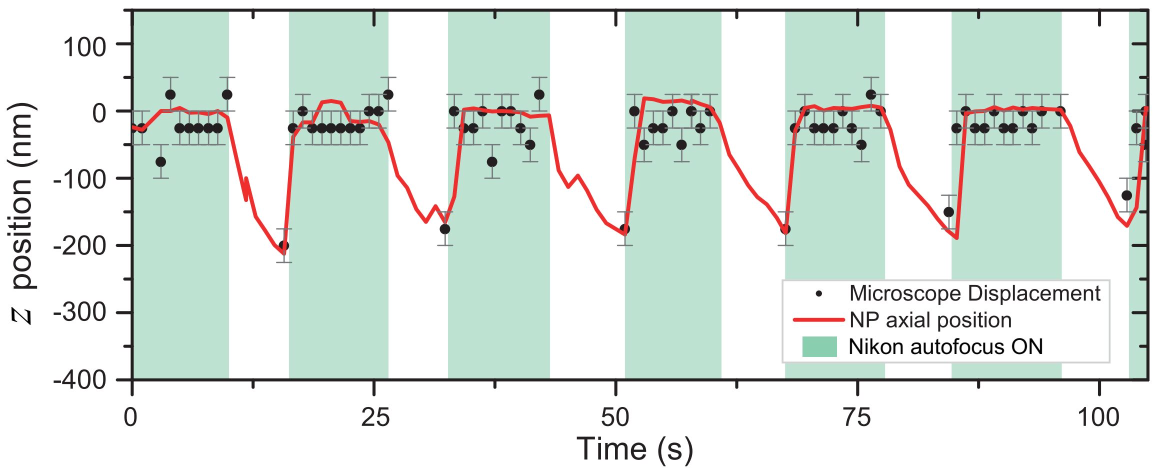

Supplementary Figure 4: Comparison of the dynamic axial accuracy of our approach with a commercial autofocus system (Nikon Perfect Focus). The autofocus system controls the microscope displacement (black dots), while our method quantifies the actual axial position of a 100 nm gold nanoparticle (red line). Emerald stripes indicate the intervals where the autofocus system is switched on (note that here the stage is heated to speed the axial drift).

Effects of optical aberrations

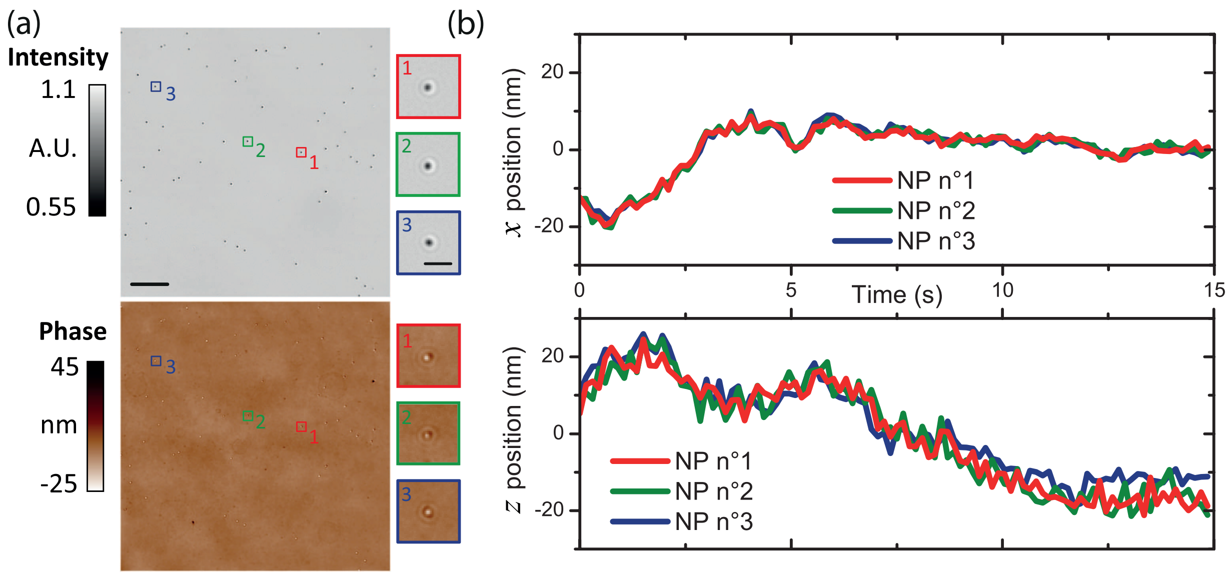

Supplementary Figure 5: (a) Intensity (top) and phase (bottom) measurement on 100 nm gold NPs at $\lambda = 560 \pm 10$ nm. Scale bar: 10 μm. Subimages : 2 μm. (b) Drifts measured as a function of time on 3 different particles indicated with color boxes and numbers on the image *(a)*. (Top) Along the x direction drift. (Bottom) Along the z direction drift.

Local surface plasmon resonance and polarization effects

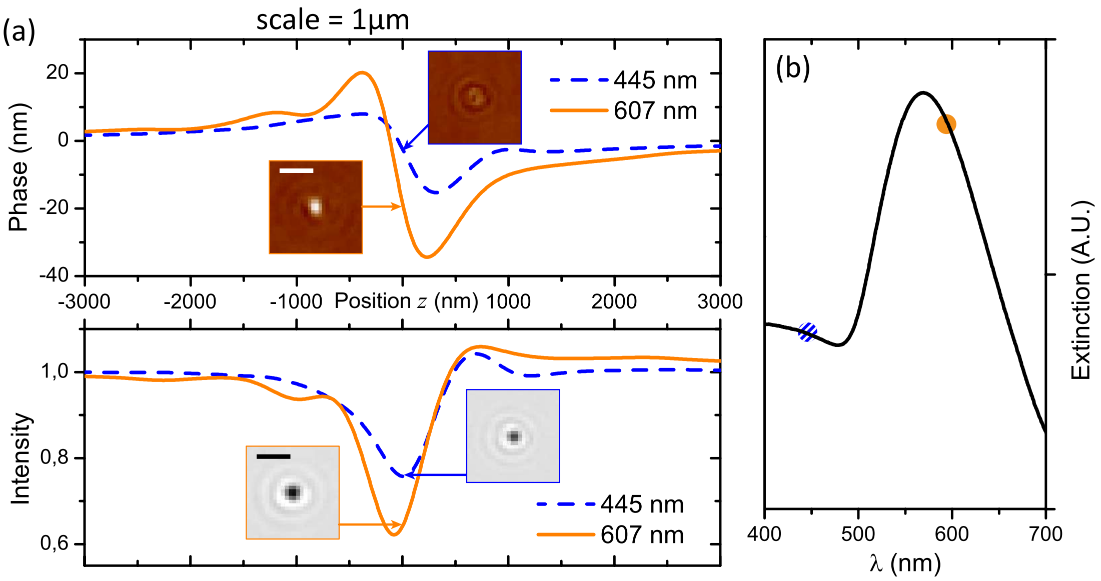

Supplementary Figure 6: (a) Through-focus Phase (top) and intensity (bottom) value measured on a 100 nm gold NP at $\lambda = 607$ nm (solid orange) and $\lambda = 445$ nm (dashed blue). Intensity and phase images acquired near the focus are represented as sub-images. Scale bars $= 1$ μm. (b) Extinction cross-section of a spherical 100 nm gold NP as a function of the wavelength (computed using the Mie theory). $\lambda = 445$ nm and 607 nm are indicated.

Comparison between metal and dielectric nanoparticles

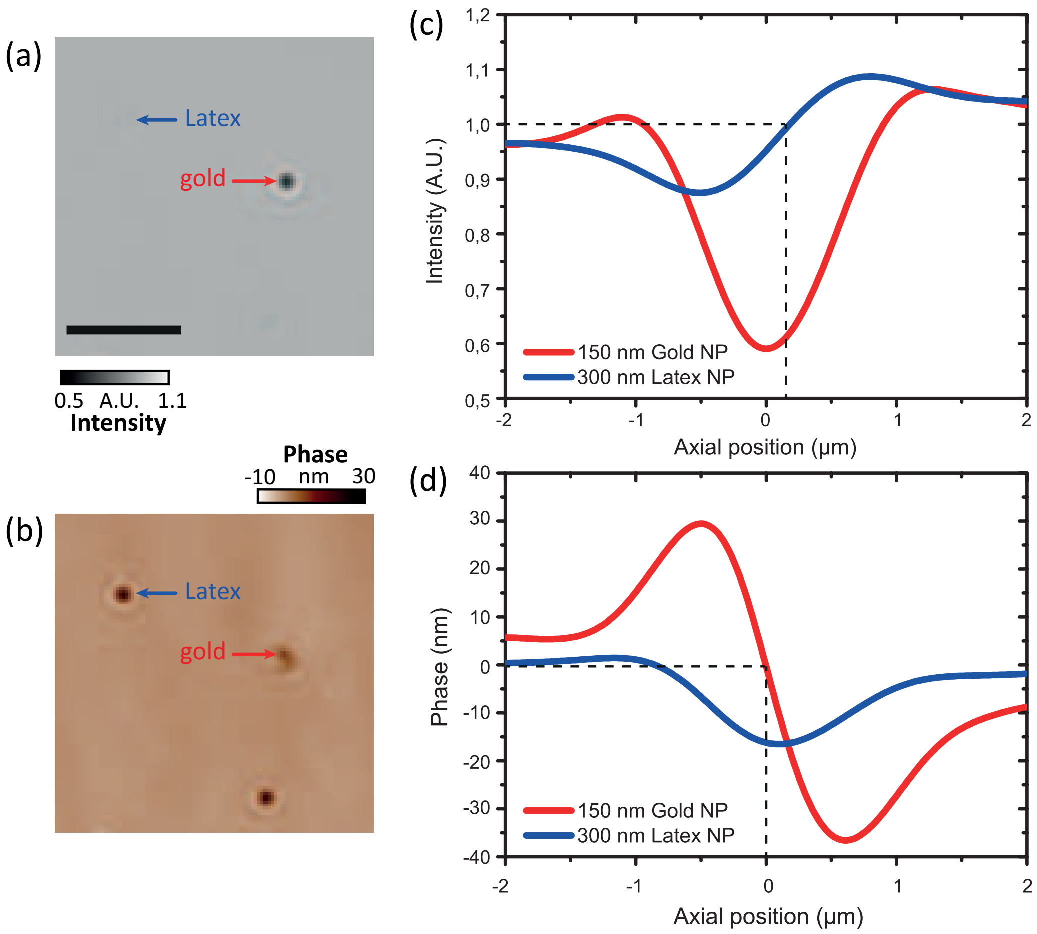

Supplementary Figure 7: Comparison between gold and polystyrene nanoparticles. (a) Intensity and (b) phase images on a mixture of 150 nm gold and 300 nm latex nanoparticles at $\lambda = 680$ nm. Scale bar: 5 μm. (c,d) Through-focus evolutions of the intensity (c) and phase (d) obtained by numerical propagation of the measurement made in *(a,b)* showing the responses of gold NP (red) and latex NP (blue).

Numerical propagation and refocusing

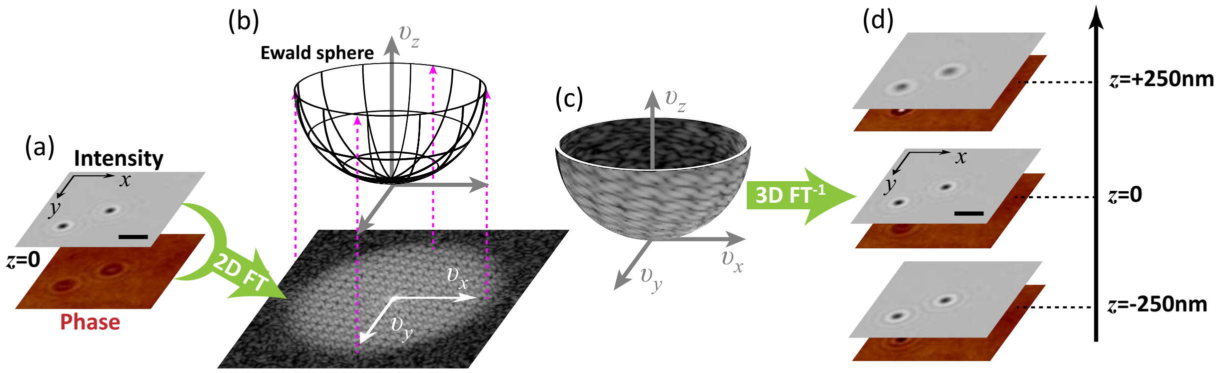

Supplementary Figure 8: (a) Example of an intensity and phase images of 100 nm gold nanobeads. (b) Top : accessible object frequency (Ewald sphere), given the optical parameters (wavelength, numerical aperture, coherent illumination). Bottom : 2D Fourier Transform (FT) in log scale of the electromagnetic field obtained from the combination of the image from *(a)*. (c) 2D FT of *(b,bottom)* projected on the Ewald sphere. (d) Intensity and phase images numerically obtained in different z planes after 3D inverse Fourier Transform ($FT^{-}1$) of *(c)*.

Supplementary tables

Theoretical description of the through-focus dependencies

Data	λ (nm)	Meas. A	Meas. $\partial\delta/\partial z$	Model. $\partial\delta/\partial z$
Suppl. Fig. 6a	445	0.24	-0.062 ± 0.003	-0.063
Suppl. Fig. 6a	607	0.38	-0.13 ± 0.02	-0.12
Fig. 1c	670	0.15	-0.033 ± 0.002	-0.035

Supplementary Table 1: Comparison of near-focus phase slope values for different wavelengths considering gold NP.

Image resolution gain with 3D drift correction

Labeling	Drift compensation	Image	Detection events (full-image)	Super-resolution in std. dev. (nm)
Tubulin	No	S.Fig. 1.b	80861	>35 (S.Fig. 2.b,pink)
	Lateral (xy)+low threshold	S.Fig. 1.c	128222	>33 (S.Fig. 2.b,yellow)
	Axial (z)	Data of S.Fig. 1	188861	>37 (S.Fig. 2.b,blue)
	3D (xyz)	S.Fig. 1.d	188861	**21**±1 (S.Fig. 2.b,green)
F-actin	No	Fig. 3.b	172615	>35 (S.Fig. 2.d,pink)
	Lateral (xy)+low threshold	Fig. 3.c	251137	>38 (S.Fig. 2.d,yellow)
	Axial (z)	Data of Fig. 3	262587	>30 (S.Fig. 2.d,blue)
	3D (xyz)	Fig. 3.d	262587	**11**±1 (S.Fig. 2.d,green)

Supplementary Table 2: Comparison of the number of detected molecules and the experimental super-resolution with tubulin or F-actin labeling and different axis stabilization.

Effects of optical aberrations

Particles implied (+ distance)	Std. dev. along x (nm)	Std. dev. along z (nm)
(1, 2) $\rightarrow$ 15 μm	0.99 ± 0.05	3.51 ± 0.16
(2, 3) $\rightarrow$ 30 μm	1.03 ± 0.05	3.81 ± 0.17
(1, 3) $\rightarrow$ 45 μm	1.06 ± 0.05	4.25 ± 0.19

Supplementary Table 3: Comparison of the difference of localization between pairs of 100 nm nanoparticles.

Supplementary Discussion

Theoretical description of the through-focus dependencies

The intensity and the phase are not independent variables respect to the imaging plane. They are indeed in quadrature, as one can observe for example on the Fig. 1c: when the slope of the intensity versus z is null, the phase versus focus is important and *vice versa*. The so-called transport-of-intensity equations can be used to describe the link between the intensity and phase as a function of the axial plane [1]. Let u be the amplitude of the EM field:

$$u\,(r,z) = \sqrt{I\,(r,z)} \cdot e^{j\varphi(r,z)}, \tag{1}$$

with r the lateral (x,y) coordinates and z the axial coordinate. I represents the EM field intensity and φ its phase. It is noteworthy that with our phase-imaging approach (QWLSI) this is actually the optical path difference (OPD, denoted δ in the further equations) that is measured. The OPD is linked to phase with:

$$\delta = \frac{\varphi_{prop} - \varphi}{k}, \tag{2}$$

with $k = 2\pi/\lambda$ the wavevector norm and φ_{prop} the propagation phase defined as $\varphi_{prop} = k \cdot z$.

Considering a free-space propagation of a coherent beam, one can write [1]

$$\left(j\frac{\partial}{\partial z} + \frac{\nabla^2}{2k} + k \right) u = 0, \tag{3}$$

with ∇ the lateral gradient operator. Combining the equ. (1) and (3) we obtain

$$e^{j\varphi}\left[\frac{j}{2\sqrt{I}} \cdot \frac{\partial I}{\partial z} - \frac{\partial \varphi}{\partial z}\sqrt{I} + \frac{1}{2k}\left(\frac{\nabla^2 I}{2\sqrt{I}} - \frac{(\nabla I)^2}{4I\sqrt{I}} + j\frac{\nabla\varphi\nabla I}{\sqrt{I}} + j\sqrt{I}\nabla^2\varphi - \sqrt{I}(\nabla\varphi)^2\right) + k\sqrt{I}\right] = 0. \tag{4}$$

Useful relations between I and φ can be reached by multiplying the equ. (4) by the complex conjugate of u^*. A second equation can be generated by taking the complex conjugate of (4)$\cdot u^*$. By adding or subtracting these two equations, simple links between the axial phase or intensity derivative and their lateral gradients can be obtained:

$$\begin{cases} (4) \times u^* - (4)^* \times u \Leftrightarrow \dfrac{\partial I}{\partial z} = -\dfrac{1}{k}\nabla\,(I\nabla\varphi) \\ (4) \times u^* + (4)^* \times u \Leftrightarrow \dfrac{\partial \varphi}{\partial z} = \dfrac{\nabla^2 I}{4kI} - \dfrac{(\nabla I)^2}{8kI^2} + \dfrac{I\nabla^2\varphi}{2kI} + k \end{cases}. \tag{5}$$

The first part of Eq.(5) is well known in the field of quantitative phase imaging as numerous techniques are actually based on this equation to retrieve the phase using intensity measurements

[2, 3, 4]. The second part is less known as it is usually simpler to measure the intensity rather than the phase. Let us use both of them in the scope of non resolved absorbing sample imaging (ex. gold NPs). In this case the in-focus intensity and phase images can be modeled as:

$$\begin{cases} I\left(r, z=0\right) = I_0 \left[1 - Ae^{-\frac{r^2}{2\sigma^2}}\right] \\ \varphi\left(r, z=0\right) = 0 \end{cases}, \tag{6}$$

considering for simplification purpose that the imaging response of the optical microscope can be described by a 2D Gaussian function with standard deviation σ. A is the maximum intensity attenuation value at the particle image center and I_0 the background intensity. As the object is purely absorbing, its phase at the plane $z = 0$ is constant equal to $\varphi_{prop} = 0$. Using the equ. (5) and (6) one can obtain:

$$\begin{cases} \frac{\partial I}{\partial z}\left(z=0\right) = 0 \\ \frac{\partial \varphi}{\partial z}\left(z=0\right) = k + \frac{A}{4k\sigma^2\left(e^{\frac{r^2}{2\sigma^2}} - A\right)}\left[1 - \frac{r^2}{\sigma^2}\left(1 + \frac{A}{2}\frac{e^{-\frac{r^2}{2\sigma^2}}}{\left(e^{\frac{r^2}{2\sigma^2}} - 1\right)}\right)\right] \end{cases}. \tag{7}$$

By considering the microscope as aberration-free with an objective numerical aperture of $\mathrm{NA}_{obj} = 1.4$, it means that $\sigma = \frac{r_{PSF}}{2\sqrt{2\ln 2}} = \frac{0.26\lambda}{\mathrm{NA}_{obj}}$. At the NP image center (*i.e.* $r = 0$) the equ. (7) can be rewritten in OPD slope:

$$\begin{cases} \frac{\partial I}{\partial z}\left(r=z=0\right) = 0 \\ \frac{\partial \delta}{\partial z}\left(r=z=0\right) \approx -\frac{A \cdot \mathrm{NA}_{obj}^2}{\pi^2\left(1-A\right)} \end{cases}. \tag{8}$$

As experimentally observed, the intensity derivative along z is null when the particle is at the focus (Fig. 1c in black). Simultaneously, the OPD derivative along z is constant confirming the linear z dependency of the measured phase around the focus (Fig. 1c in red). An interesting point is that the OPD slope is independent of λ, the key parameter being the attenuation A: it means that our technique can be used at any wavelength only choosing a NP that exhibits a strong attenuation at this particular wavelength.

Moreover, the phase slope values (near the focus) retrieved with this model are in good agreement with the measured ones (Suppl. Tab. 1).

Image resolution gain with 3D drift correction

The lateral super-resolution for pointillist techniques depends on multiple aspects: (i) the lateral drifts during the acquisition, (ii) the number of detected molecules (Nyquist criterion), and (iii) the localization precision which is mainly affected by the fluorophore brightness. We infer the spatial resolution in dSTORM imaging by measuring the histogram of the detected positions along a single nanostructure. Tubulin and actin filaments are good candidates, and are commonly used to determine the resolution in super-resolution experiments [5, 6, 7]. One limitation of this method is that the measured histogram width is a convolution of the object by the microscope point spread function. Hence, the histogram width depend on the size of the object. Typically, the smallest structures with isolated F-actin filaments labeled using phalloidin have a diameter below 10 nm, while tubulin filaments labeled with primary and secondary anti-bodies have a diameter around 50 nm. Therefore, the histogram widths and standard deviations measured on F-actin filaments are narrower than those obtained on tubulin.

Suppl. Figure 2 and Suppl. Table 2 show the results obtained using the data from the experiments described in Fig. 3 and Suppl. Fig. 1. The lateral resolution is significantly better with (x, y, z) drift compensation up to a factor of 3 in our F-actin experiment. The lateral resolution, in terms of standard deviation of the position histogram, is measured to be 11$\pm$1 nm with F-actin labeled with phalloidin plus 3D drift compensation, while the resolution for the tubulin labeling experiment and 3D drift correction is 21$\pm$1 nm. In addition to the lower spatial resolution when no drift compensation is active, the number of detected molecules is also 3 times lower as compared to a full-3D drift compensation. This comes as the defocus accumulated during the experiment blurs the fluorescence intensity below the detection threshold. Decreasing the detection threshold in the absence of axial stabilization helps to increase the number of detected molecules. However, as the detection signal-to-noise ratio is low for many fluorophores, it leads to a high localization uncertainty and thus a bad lateral resolution although (x, y) drift correction is applied.

Axial localization accuracy as compared to commercial autofocus system

We concentrate here on the axial localization dynamics and compare our method with a commercial autofocus system (Perfect Focus, Nikon, Japan) which monitors the reflection of infrared light at the coverslip surface. The single output from this commercial solution is the actual microscope axial position with an accuracy of 25 nm. Therefore, to test our technique, we decided to sequentially activate and deactivate the autofocus system while measuring with our approach the relative axial displacement of a 100 nm gold nanoparticle deposited on a coverslip and immersed in water (observation wavelength 610 nm). In order to increase the drift speed, the sample was heated using a commercial sample holder (Tokai Hit, Tokai, Japan).

Suppl. Figure 4 shows the result of this comparison. When the commercial autofocus is on (emerald stripes), our technique measures a residual nanoparticle axial drift that is not detected with the autofocus, or improperly corrected (see the dispersion of black dots when the autofocus is on as compared to the actual nanoparticle position). When the autofocus is turned off (white stripes), the focus drift is no longer compensated by the microscope mechanical displacement. When the autofocus is turned on again, the microscope makes a displacement whose amplitude is correct with the measured nanoparticle axial drift. In all cases, it appears that our localization approach provides a better spatial and temporal accuracy. Moreover, we point out that autofocus systems based on the reflection of light at the coverslip/medium interface are inefficient in the case of index matching between the coverslip and the medium (as for example for in-depth STED measurements), which is not the case for our technique.

Effects of optical aberrations

In the theoretical description of the through-focus intensity and phase imaging (Section 3), we have considered the microscope objective as being perfectly corrected from optical aberrations. This assumption is used to simplify the theoretical treatment, but it is not a necessary condition for our technique. Especially at high numerical aperture such as 1.4 or even 1.49 used for super-resolution imaging, the microscope objective might have optical aberrations. One of the major advantage of our technique is that there is no assumption on the optical response of the imaged nanoparticles. As both the intensity and the phase are simultaneously measured, the optical aberrations are directly taken into account in the numerical propagation algorithm. High optical aberrations will only result in a drop in our positioning sensitivity, but measurements are still possible.

Figure 2.b,c and Movie 1 illustrate the accuracy of refocusing with large defocus and thus spherical aberrations. For a defocus of several microns, the refocused intensity PSF is enlarged which leads to a reduction of lateral localization precision, and the trough-focus phase slope is decreased leading to a drop in axial localization sensitivity. Remarkably, this drop in localization accuracy remains limited, even for defocus much larger than the depth-of-field. For a 3 μm defocus, we typically achieve lateral precision below 10 nm and axial precision below 40 nm (Fig. 2c).

Within the wavefront sensor field of view (FoV), one way to experimentally evaluate the influence of field aberrations is to compare the 3D localization of three different immobilized particles: 2 near the center of the FoV (particle 1 and 2) and 1 near the edge (particle 3), as presented in Suppl. Fig. 5. The particles 1 and 2 are distant of about 15 μm, while the particles 1 and 3 are distant of 45 μm and the particles 2 and 3, 30 μm. Suppl. Table 3 summarizes the standard deviation for 100 measurements of the difference of positioning for each couple of particles, along both the lateral x and the axial z direction. There is a small effect of the field aberrations that tends to slightly decrease the measurement precision on the edge of the FoV. However, this variation is very limited (less than 1 nm) and can be safely neglected. This shows that any nanoparticle(s) within the FoV can be considered to stabilize the setup.

Local surface plasmon resonance and polarization effects

To optimize the accuracy of our 3D position retrieval technique, it is useful to maximize the interaction of light with the nanoparticle. This can be elegantly performed by working close to the local surface plasmon resonance (LSPR) of the nanoparticle [8]. Near the LSPR, the extinction cross-section is remarkably high due to the light that is resonantly absorbed and scattered by the nanoparticle. One consequence is that the through-focus intensity and phase responses are respectively even and odd with important slopes and extremum values near the LSPR wavelength. Suppl. Figure 6a shows through-focus intensity and phase values for 100 nm gold NP at two different wavelengths $\lambda \approx 445$ nm and $\lambda \approx 607$ nm, respectively off and near resonance conditions. Using numerical computations according to the Mie theory, the extinction cross section of a 100 nm gold sphere exhibits a resonance around $\lambda = 580$ nm (Suppl. Fig. 6b). This is visible on the through-focus intensity and phase responses: the curves are more modulated at 607 nm (solid orange line in Suppl. Fig. 6a) compared to 445 nm (dashed blue line in Suppl. Fig. 6). Our 3D superlocalization approach is thus more sensitive for this kind of particle around 580 nm.

In this study, we use spherical nanoparticles and non-polarized illumination from a collimated halogen lamp. As the nanoparticles are isotropic, the illumination polarization has no influence on our results. However, if non 3D isotropic particles such as metal nanorods were used for our 3D localization approach, the illumination polarization would need to be tuned to maximize the sensitivity of our technique. This is due to the enhancement of interaction between the nanoparticle and the illumination light.

Comparison between metal and dielectric nanoparticles

So far, we considered 3D super-localization of gold nanoparticles which have a high extinction, and whose optical response is dominated by the imaginary part of the gold refractive index. To generalize our approach, we investigate here dielectric nanoparticles (such as latex or silica NPs) where the optical response primarily depends on the real part of the particle refractive index. Suppl. Figure 7 shows a comparison between gold and latex nanoparticles. At the microscope focus, the latex nanoparticles are almost invisible in intensity while they largely stand out of the background in the phase image. Therefore the phase image can be used to laterally localize the latex nanoparticles, while the intensity variation can be used to obtain the axial position. Interestingly, the shapes of the phase and intensity axial responses are inverted between metal and dielectric nanoparticles (Suppl. Fig. 7c,d). While gold nanoparticles provide a sharper response due to their higher extinction, our technique can nevertheless be applied to dielectric nanoparticles. Notice also that the two nanoparticles are localized by our technique in different planes distant of 150 nm. This is consistent with the fact that the latex particles are larger than the gold ones by 150 nm.

Supplementary Methods

Numerical propagation and refocusing

As the illumination can be considered as a non-polarized plane-wave, it is possible to numerically propagate an acquired scalar electromagnetic field in different planes. It generates images in various planes without any moving elements. Indeed, under coherent illumination, the sample imaged frequencies K_o are carried by a portion of the Ewald sphere [9, 10]. If the illumination wavevector $\overrightarrow{k_i}$ is collinear to the optical axis, then it is straightforward to obtain a representation of K_o in a trans-illumination scheme, given the fact that diffraction is an elastic process:

$$\begin{cases} \overrightarrow{k_d} = \overrightarrow{k_i} + \overrightarrow{K_o} \\ \left\|\overrightarrow{k_d}\right\| = \left\|\overrightarrow{k_i}\right\| \end{cases}, \\ \Rightarrow K_{oz} = -k_i + \sqrt{k_i^2 - \left(K_{ox}^2 + K_{oy}^2\right)}, \tag{9}$$

with $\overrightarrow{k_d}$ a diffracted wavevector.

A phase and intensity measurement in a single 2D plane (ex. Suppl. Fig. 8a) is enough to regenerate the 3D frequency space (Suppl. Fig. 8c) by projecting the frequency content of the measured 2D electromagnetic field onto the 3D Ewald sphere (Suppl. Fig. 8b). This allows to retrieve the phase and the intensity images in different planes by a simple inverse Fourier Transform (FT).

Accuracy measurement protocol

Each measurement p of the nanoparticle position is the sum of the sample displacement d plus the error of our approach ε. In order to measure the accuracy of our approach, that is the standard deviation of the error $\sigma(\varepsilon)$, we performed 100 repeated measurements of the nanoparticle position at 50 Hz rate. The total 2s integration time implies that during the recording some technical noise will also affect the sample displacement d, which is not a constant. To assess only the accuracy of our superlocalization method $\sigma(\varepsilon)$, we subtract the measured position of two immobilized nanoparticles visible within the same image (Fig. 1b). This removes the correlated noise $\sigma(d)$ of the actual sample displacement leaving only the combined noise of each localization measurement:

$$\begin{cases} p_1 = d + \varepsilon_1 \\ p_2 = d + \varepsilon_2 \end{cases} \\ \Delta p = p_1 - p_2 = \varepsilon_1 - \varepsilon_2, \tag{10}$$

with p_i, ε_i and d respectively the ith particle measured position, the error and the real drift. As the position measurement of each particle is driven by independent but equivalent noise, it means

that the mean square fluctuation of each particle $\sigma(p_i) = \sigma(\epsilon_i)$ is the same for each particle (*i.e.* $\sigma(\epsilon_1) = \sigma(\epsilon_2)$) and thus:

$$\sigma(\varepsilon) = \frac{\sigma(\Delta p)}{\sqrt{2}}. \tag{11}$$

With the technique applied on 100 nm gold nanobeads at $\lambda = 594$ nm, the accuracy from the comparative positions of two nanoparticles has been found to $\sigma(\varepsilon_{xy}) = 0.7$ nm in the lateral directions and $\sigma(\varepsilon_z) = 2.7$ nm in the axial direction when the particle is near the focus.

It is interesting to note that this approach allows to quantify the fluctuations of the setup stability $\sigma(d)$ on a 2 seconds timescale:

$$\sigma^2(d) = \sigma^2(p) - \sigma^2(\varepsilon). \tag{12}$$

Using the data in Fig. 2b for $\sigma(p)$ and the noise estimates for $\sigma(\varepsilon)$ from the correlated positions of 2 nanoparticles, we obtain the setup lateral stability $\sigma(d_{xy}) = 1.3$ nm while the axial stability is $\sigma(d_z) = 6$ nm. These fluctuations originate mainly from the noise of the piezo stage and vibrations from the wavefront sensing camera. Both are independent of our conceptual approach.

Preparation of dSTORM samples

CHO cells were grown in Dulbecco's Modified Eagle Medium (DMEM) supplemented with 10% Fetal Bovine Serum (FBS), 1% L-Glutamin and 1% penicillin/streptomycin (Life Technologies, U.S.A.) in a cell humidified culture incubator (37°C and 5% CO_2). After several days, cells were plated at low confluency coverslips previously coated with gold NPs and a poly-L-lysine layer. A fixation and a staining step have been performed. Prior to fixation, all chemical reagents were pre-warmed at 37°C. 24h after plating, cells were washed 3 times using PHEM buffer (60 mM PIPES, 25 mM HEPES, 5 mM EGTA and 2 mM Mg acetate adjusted to pH 6.9 with 1 M KOH), pre-extracted for 1 min in 0.5% Triton X-100 (Sigma-Aldrich, U.S.A.) and fixed for 20 min in 4% paraformaldehyde, 0.02% glutaraldehyde and 0.5% Triton, then washed 3 times in phosphate buffered saline (PBS) (Sigma-Aldrich, U.S.A.). The samples were post-fixed 10 min with PBS 0.1% Triton, reduced for 10 min with $NaBH_4$ and washed again in PBS.

At this step, we have two labeling protocols either for microtubule or F-actin labeling. For microtubule labeling, cells are blocked for 15 min in PBS 1% Bovine Serum Albumin (BSA), before incubating for 1 hour at room temperature with 1:1000 mouse alpha-tubuline antibody (T6199, Sigma-Aldrich, U.S.A.) in 0.1% BSA diluted in PBS, followed by 3 washes in PBS and incubated for 45 min with a 1:400 goat anti-mouse Alexa Fluor 647 (A-21237, molecular probes, invitrogen, U.S.A.) diluted in PBS 0.1% BSA. Three additional washes are done in PBS. The second staining used a high-affinity F-actin probe: the phalloidin-Alexa Fluor 647 (A-22287 molecular probes, invitrogen, U.S.A.). Cells were pre-incubated for 20 min in PBS 1% BSA prior to add the phallotoxin staining solution for 20 min at room temperature. The samples were then washed in PBS and conserved in PBS before mounting in a dSTROM buffer for observation.

Super-resolved image acquisition and reconstruction

We acquired 2000 to 3500 blinking images for each super-resolved reconstructed image. The axial defocus is compensated in real time, by moving the objective, using the z displacement measured with our approach. On each image, a local threshold τ is applied to roughly detect each point spread function (PSF). Each PSF is then fitted with a Gaussian curve using the Levenberg-Marquardt non-linear algorithm on a home-made Labview software (LV2011, National Instrument, USA) to measure the particle centroid. The lateral drift is compensated after the acquisition by subtracting the measured lateral displacement of the reference NP to each measured centroid. In order to take into account the signal-to-noise ratio of each detected fluorophore, the super-resolved image is created by adding a 2D Gaussian function centered on each detected particle centroid, with a standard deviation depending on the fluorescence signal-to-noise ratio (SNR) for this particle.

To generate the super-resolved images when no axial stabilization is applied (ex. Fig. 3b,c or Suppl. Fig. 1b,c), the same set of camera images and thus detected particles are used. Prior to the dSTORM experiments, the microscope PSF has been calibrated by acquiring images of individual fluorescent nanobeads (50 nm diameter) and measuring the signal variation as a function of the axial defocus. The effects of the sample axial drift without axial stabilization are obtained as follows: (i) each fluorophore image position, energy and background is measured *via* 2D Gaussian fitting on the acquired stabilized image. (ii) A new fluorophore subimage is then numerically generated for each detected molecule, respecting the energy conservation, using the experimental microscope PSF corresponding to the total axial defocus that would have been present in the absence of stabilization. (iii) The background is added to the subimage and Poisson noise is computed on each pixel given this new value and the gray-level to photon conversion factor of the camera. (iv) If some pixels in the generated final subimage is above the threshold τ, the particle is still considered as detected and its 2D localization is computed. (v) The super-resolved image without axial defocus is generated as previously described, by the addition of each detected molecule.

Supplementary References

[1] Michael Reed Teague. Image formation in terms of the transport equation. *J. Opt. Soc. Am. A*, 2(11):2019, 1985.

[2] A. Barty, K. A. Nugent, D. Paganin, and A. Roberts. Quantitative optical phase microscopy. *Opt. Lett.*, 23(11):817–819, 1998.

[3] Shan Shan Kou, Laura Waller, George Barbastathis, and Colin J. R. Sheppard. Transport-of-intensity approach to differential interference contrast (ti-dic) microscopy for quantitative phase imaging. *Opt. Lett.*, 35(3):447–449, Feb 2010.

[4] Kevin G. Phillips, Steven L. Jacques, and Owen J. T. McCarty. Measurement of single cell refractive index, dry mass, volume, and density using a transillumination microscope. *Phys. Rev. Lett.*, 109:118105, Sep 2012.

[5] Bo Huang, Sara A Jones, Boerries Brandenburg, and Xiaowei Zhuang. Whole-cell 3d storm reveals interactions between cellular structures with nanometer-scale resolution. *Nature methods*, 5(12):1047–1052, 2008.

[6] Gleb Shtengel, James A Galbraith, Catherine G Galbraith, Jennifer Lippincott-Schwartz, Jennifer M Gillette, Suliana Manley, Rachid Sougrat, Clare M Waterman, Pakorn Kanchanawong, Michael W Davidson, et al. Interferometric fluorescent super-resolution microscopy resolves 3d cellular ultrastructure. *Proceedings of the National Academy of Sciences*, 106(9):3125–3130, 2009.

[7] Yina Wang, Joerg Schnitzbauer, Zhe Hu, Xueming Li, Yifan Cheng, Zhen-Li Huang, and Bo Huang. Localization events-based sample drift correction for localization microscopy with redundant cross-correlation algorithm. *Opt. Express*, 22(13):15982–15991, Jun 2014.

[8] Lukas Novotny and Bert Hecht. *Principles of nano-optics*. Cambridge university press, 2006.

[9] Yann Cotte, Fatih Toy, Pascal Jourdain, Nicolas Pavillon, Daniel Boss, Pierre Magistretti, Pierre Marquet, and Christian Depeursinge. Marker-free phase nanoscopy. *Nat. Photon.*, 7(2):113–117, feb 2013.

[10] Pierre Bon, Sherazade Aknoun, Serge Monneret, and Benoit Wattellier. Enhanced 3d spatial resolution in quantitative phase microscopy using spatially incoherent illumination. *Opt. Express*, 22(7):8654–8671, Apr 2014.
